# Union by the First Intention

**Published:** 1847-11

**Authors:** 


*Union by the First Intention.*—In alluding to union by the first intention, when treating of amputation of the thigh, M. Sedillot says:

The most exact approximation cannot prevent an excavation which serves as a reservoir for the fluids exuding from the wound. A pultaceous, grayish matter, a species of false membrane, formed of the fibrous portion of the blood, which should be eliminated, is retained within the wound, and converted into a purulent and fetid sanies, which may give rise to severe consequences, such as phlebitis, purulent infection, etc.; the bone, macerated in the pus, becomes denuded of the periosteum and necrosed; all this while adhesion of the skin, which is easily produced, masks what is going on in deep-seated parts, until tension, tumefaction and heat reveal it. Unless incisions are practiced, or the cicatrization torn open, collections of matter are formed, and the *morale* of the patient—who, believing himself nearly cured, finds that all is well nigh to suffer again—becomes injuriously influenced.

More security attends a different practice. After the ligature of the vessels, the blood is to be carefully stanched, and the wound is neither to be closed by strapping nor crammed with lint. A barrel compress spread with cerate and a few pieces of fine lint are alone to be applied, the muscles of the limb being supported by a roller. During the first days the surface of the wound becomes covered with a grayish pultaceous crust, which, however, is soon detached, leaving red, healthy granulations, the precursors of cicatrization. The fears of the patient of the first dressing have been especially objected to this plan; but such objection is only valid when the dressing is performed prior to the complete establishment of suppuration, and ceases to be so if this be delayed to the fourth or fifth day, when the compresses are nearly detached by the suppuration, and their removal gives relief instead of pain to the patient. After this the wound should be dressed daily, for frequent dressings are the best means for the prevention of danger from the sojourn of pus.—*Medico-Chir. Review.*

